# The cyclin-dependent kinase inhibitor flavopiridol (alvocidib) inhibits metastasis of human osteosarcoma cells

**DOI:** 10.18632/oncotarget.25239

**Published:** 2018-05-04

**Authors:** Loredana Zocchi, Stephanie C. Wu, Jie Wu, Ken L. Hayama, Claudia A. Benavente

**Affiliations:** ^1^ Department of Pharmaceutical Sciences, University of California, Irvine, CA 92697, USA; ^2^ Department of Developmental and Cell Biology, University of California, Irvine, CA 92697, USA; ^3^ Department of Biological Chemistry, University of California, Irvine, CA 92697, USA; ^4^ Chao Family Comprehensive Cancer Center, University of California, Irvine, CA 92697, USA; ^5^ Department of Microbiology and Molecular Genetics, University of California, Irvine, CA 92697, USA

**Keywords:** osteosarcoma, flavopiridol, metastasis, migration, cancer

## Abstract

Osteosarcoma is the most common primary malignant neoplasm of bone and typically occurs in children and young adults. As a highly metastatic malignancy, 15–20% of osteosarcoma patients are diagnosed after the tumor has already metastasized (typically to the lungs), which translates to 5-year survival rates of <40%. Here, we tested the effect of the cyclin-dependent kinase (CDK) inhibitor flavopiridol (alvocidib) in U2OS, SaOS-2, SJSA-1, and 143B osteosarcoma tumor cells *in vitro* and *in vivo*. Our results show that flavopiridol can drastically decrease survival in these osteosarcoma cell lines at nanomolar concentrations and induce mitotic catastrophe in p53-null osteosarcomas. We also performed transcriptome analysis (RNA-seq) of flavopiridol-treated osteosarcoma cells, which revealed significant changes in genes coding for proteins involved in cell-cell and cell-matrix adhesions, including cadherin 3 (CDH3) and 4 (CDH4). These transcriptional changes translated to a striking reduction in the ability of osteosarcoma cells to migrate and invade *in vitro*. Further, *in vivo* assessment of the effects of flavopiridol on osteosarcoma metastasis resulted in a significant reduction in the number of lung metastases in mice treated with flavopiridol at concentrations that are physiologically tolerable. This study suggests that flavopiridol, likely in combination with other cytotoxic chemotherapeutic agents, may be a promising drug for the treatment of osteosarcoma.

## INTRODUCTION

Osteosarcoma is the most common primary malignant neoplasm of the bone. It typically occurs in children and young adults, but can affect people of all ages [1]. It is derived from primitive bone-forming mesenchymal cells and frequently arises in the metaphyses of long bones [1, 2]. Despite the advances in chemotherapy and aggressive surgical resection (required for cure), the poor response to chemotherapy is still a critical factor in osteosarcoma patients [3, 4]. As a consequence, clinical outcomes for osteosarcoma patients have not substantially improved in over 30 years. The 5-year overall survival rate has remained stable at ~65% in case of local disease and <40% for patients with metastatic disease [5]. Current osteosarcoma treatments comprise a combination of methotrexate, doxorubicin, and cisplatin (a combination referred to as MAP) and occasionally ifosfamide, to induce tumor necrosis [6]. The degree of necrosis induced in the primary tumor is directly correlated with clinical outcome. Unfortunately, approximately 20% of patients relapse even when a high degree of necrosis has been achieved. A contributing factor in the resistance to cell death observed in these patients is the overexpression of anti-apoptotic members of the apoptosis regulator Bcl-2 (BCL-2) family, which includes the myeloid cell leukemia-1 protein (MCL-1) [7]. In addition to chemoresistance, the high rate of metastasis associated with this disease presents an additional therapeutic challenge. These facts highlight an urgent need for the identification of novel targeted therapies for osteosarcoma.

**Figure 1:**
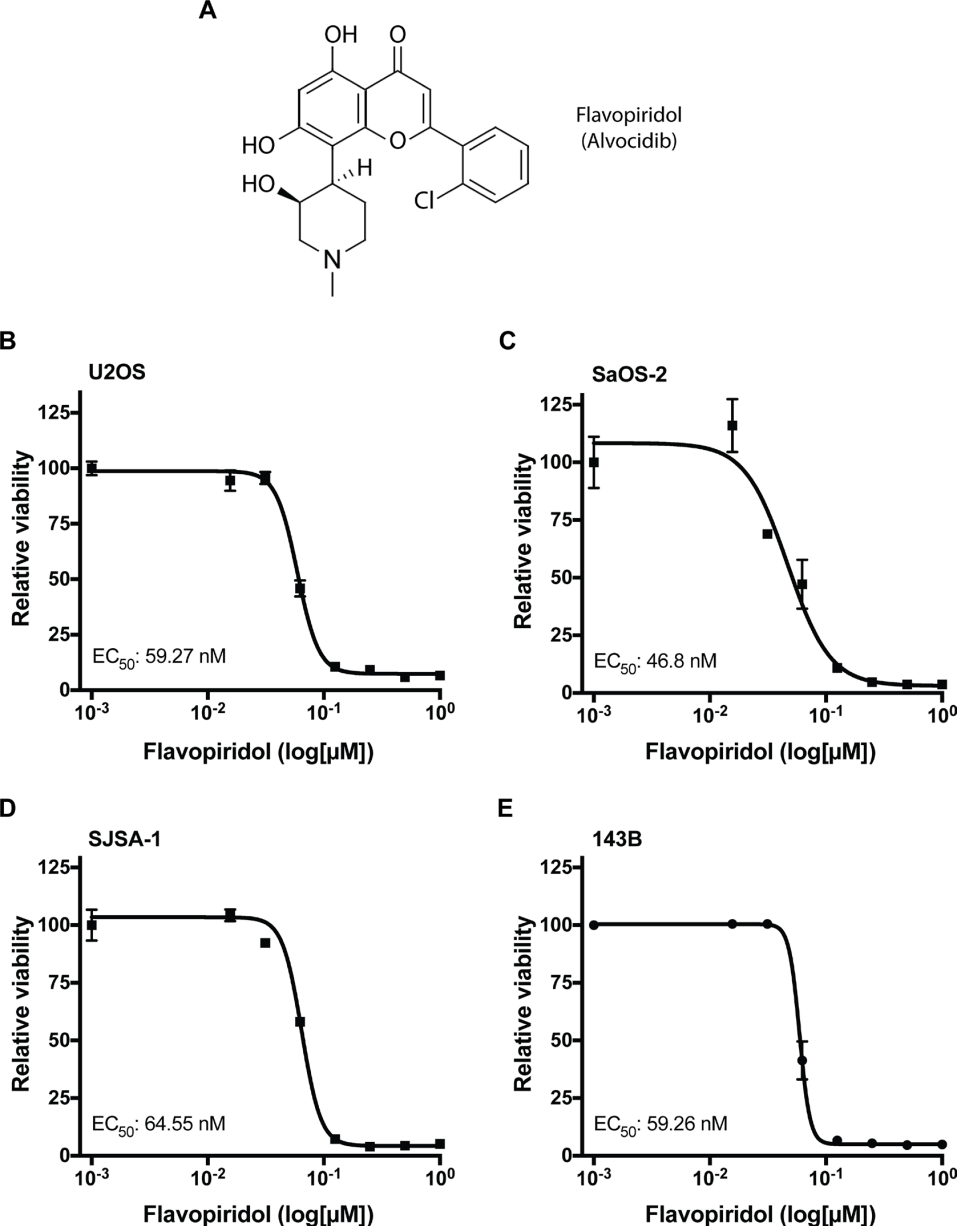
Osteosarcoma cells are sensitive to flavopiridol (**A**) Chemical structure of flavopiridol (alvocidib). (**B–E**) Dose response for flavopiridol in U2OS (B), SaOS-2 (C), SJSA-1 (D) and 143B (E) human osteosarcoma cells. Each data point is mean ± s.d. of triplicate samples. Half-maximal effective concentration (EC_50_) shown for 72 h treatment.

Recent studies evaluating cyclin-dependent kinase (CDK) inhibitors suggest that these compounds may be effective new strategies for treatment of osteosarcoma [8]. Flavopiridol (Alvocidib; Figure 1A), a semisynthetic flavonoid derived from a plant indigenous to India, is a CDK inhibitor that competes with ATP for the kinase activity site [9]. Flavopiridol induces apoptosis in chronic lymphocytic leukemia (CLL) and acute myeloid leukemia (AML) cells *in vitro* with a mechanism of action that is independent of *TP53* [10, 11]. This is important given that most osteosarcoma tumors exhibit p53 abnormalities [12, 13]. Further, flavopiridol has shown promising activity in pre-clinical and clinical trials [14, 17]. Flavopiridol is considered a broad CDK inhibitor, effective in decreasing the activity of CDK1, CDK2, CDK6, CDK7, and CDK9 [18]. Previous studies in CLL and other leukemia indicate that flavopiridol mediates its cytotoxic effects through inhibition of CDK9 and CDK7, hence hampering global RNA transcription [19, 20]. These two CDKs, are responsible for the phosphorylation of the C-terminal domain of the largest subunit of RNA polymerase II, an essential activity for both transcriptional initiation and elongation [21]. This event is also associated with a reduced level of the anti-apoptotic BCL-2 protein, MCL-1. A consequence of the reduced MCL-1 protein level is the induction of apoptosis [18]. In most studies comprising solid tumors, the reported anti-tumoral activity associated with flavopiridol has centered in its anti-proliferative and cytotoxic actions.

In this study, we examined the effects of flavopiridol treatment of four human osteosarcoma cell lines with broad genetic background: U2OS, SaOS-2, SJSA-1, and 143B. Our results suggest that flavopiridol treatment is cytotoxic at the nanomolar range in all osteosarcoma cell lines analyzed and can effectively decrease the expression of several anti-apoptotic BCL-2 family members, including MCL-1. We centered our research on the early changes in cell cycle distribution, apoptosis, gene expression, migration and metastasis following flavopiridol treatment. Interestingly, we found that flavopiridol significantly alters the expression of genes involved in cellular adhesion, leading to suppression of cell migration and invasion in osteosarcoma cell lines *in vitro* and metastasis *in vivo*. Altogether, our results suggest that flavopiridol may be useful for the treatment of osteosarcoma.

## RESULTS

### Flavopiridol decreases the viability of osteosarcoma cell lines

Previous studies have shown that flavopiridol can effectively decrease proliferation and induce apoptosis in several types of cancer [11, 22, 23]. To determine whether flavopiridol can inhibit growth and decrease cell viability in osteosarcoma, we examined four different human osteosarcoma cell lines: U2OS, SaOS-2, SJSA-1, and 143B. U2OS is a cell line derived from a moderately differentiated sarcoma of the tibia of a 15 year-old female which expresses insulin-like growth factor I and II [24]. SaOS-2 is a retinoblastoma-associated gene (*RB1)*-null and *TP53*-null cell line derived from an osteosarcoma tumor treated with RTG, methotrexate, adriamycin, vincristine, cytoxan, and aramycin-C from an 11 year-old female [25]. SJSA-1 was derived from an osteosarcoma of the femur with MDM2 proto-oncogene (*MDM2*) and GLI family zinc finger *1* (*GLI1*) gene amplification from a 19 year-old male patient. 143B is a *TP53*-mutant and is a thymidine kinase-negative osteosarcoma cell line derived from a 13 year-old female. Interestingly, regardless of genetic background, treatment of flavopiridol as a single-agent significantly decreased the viability of all four osteosarcoma cell lines in a dose-dependent manner in the nanomolar range with half-maximal effective concentration (EC_50_) values of 47–65 nM after 72 h of treatment (Figure 1B–1E).

### BCL-2 family proteins are decreased by flavopiridol treatment in osteosarcoma

**Figure 2:**
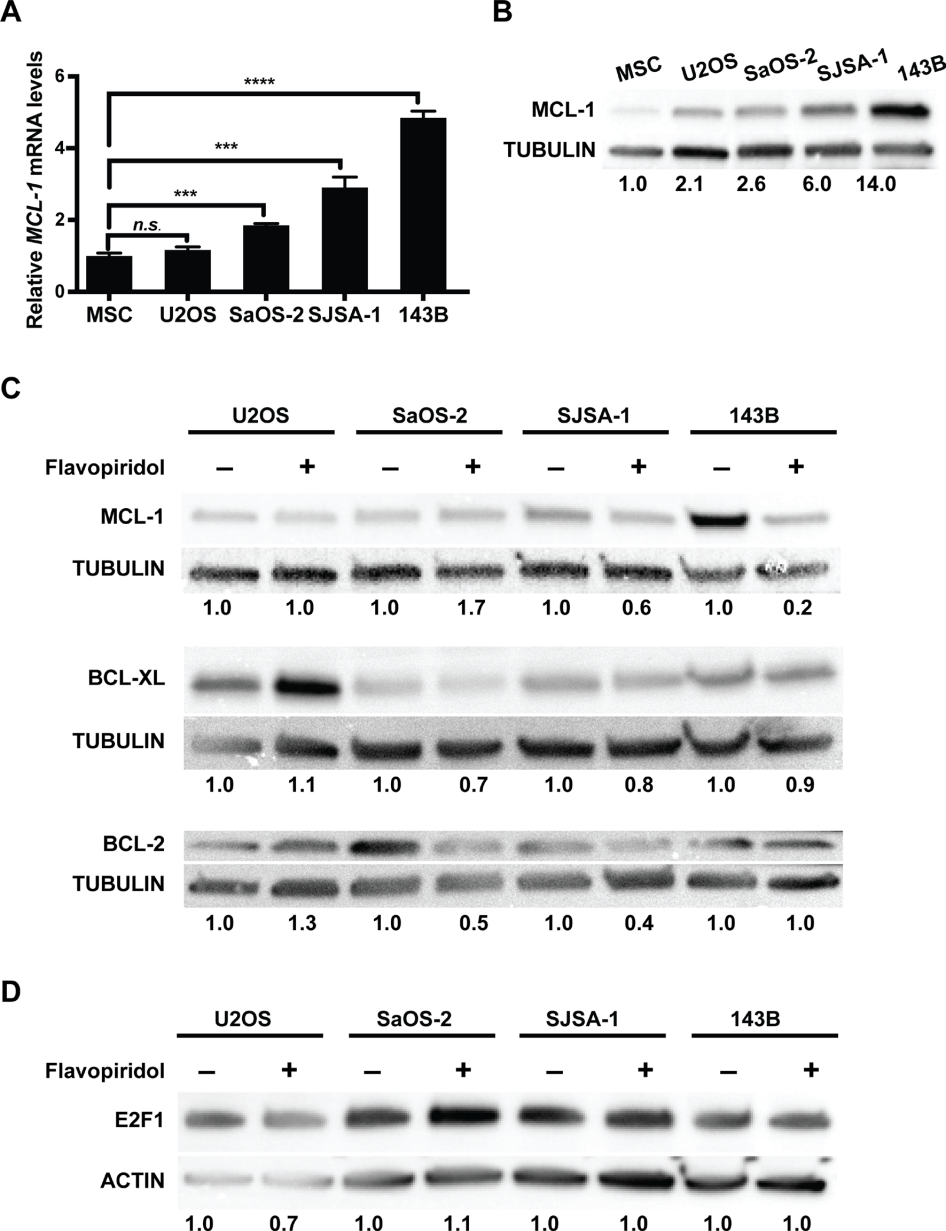
Flavopiridol alters the expression of BCL-2 family members in osteosarcoma (**A–B**) Basal MCL-1 expression in different osteosarcoma cell lines. (A) RT-qPCR analysis of *MCL-1* mRNA expression normalized to mesenchymal stem cells (MSC). (B) Western blot analysis of MCL-1 expression in U2OS, SaOS-2, SJSA-1 and 143B. Tubulin used as loading control. Band intensities were quantified by densitometry, normalized to MSC. (**C–D**) Osteosarcoma cells were harvested 16 h after 150 nM flavopiridol treatment. (C) Western blot detection of BCL-2 and BCL-2 family members MCL-1 and BCL-XL show reduced protein levels in the BCL-2 family in SaOS-2, SJSA-1, and 143B, but not U2OS after treatment. Tubulin used as loading control. Band intensities were quantified by densitometry, normalized to untreated controls. (D) Western blot detection of E2F1 shows no changes after treatment in all of the osteosarcoma cell lines. Actin used as loading control. Band intensities were quantified by densitometry, normalized to untreated controls.

Previous reports indicate that flavopiridol may decrease cell viability through the induction of apoptosis mediated by a decrease in the protein levels of MCL-1 [10, 18, 26–28]. A proposed mechanism of action suggested that flavopiridol could increase E2F transcription factor 1 (E2F1) expression and that E2F1 acted as a transcriptional repressor at the *MCL-1* promoter. Consequently, increased E2F1 protein levels following flavopiridol treatment resulted in a decrease in *MCL-1* transcription and protein levels [29, 31]. To determine if changes in E2F1 and MCL-1 proteins participate in the reduced viability that we observe in osteosarcoma cell lines following flavopiridol treatment, we analyzed the expression of these proteins. We first determined whether MCL-1 mRNA and protein baseline levels are overexpressed in untreated cells using actively dividing normal mesenchymal stem cells (MSC) as control (Figure 2A–2B). We found that SaOS-2, SJSA-1, and 143B osteosarcoma cell lines have significantly increased *MCL-1* mRNA levels when compared to MSC (Figure 2A). *MCL-1* mRNA levels were relatively homogenous, differing by a median of 1.3 ± 0.9-fold. This increased transcription translated to higher levels of the ~40 kD anti-apoptotic isoform of MCL-1, ranging from 2.6- to 14-fold increase protein expression (Figure 2B). While U2OS cells did not show a significant increase in *MCL-1* mRNA levels (Figure 2A), they did show a ~2-fold increase in MCL-1 protein (Figure 2B). This indicates that the increased MCL-1 protein levels in U2OS, and perhaps also in other osteosarcoma cells, may be a result of protein stabilization and decreased degradation. Consistent with previous reports, treatment with 150 nM flavopiridol for 16 h led to a 1.7- and 5-fold decrease of MCL-1 protein levels in SJSA-1 and 143B, respectively (Figure 2C). However, no significant changes in MCL-1 protein levels were observed in U2OS and SaOS-2 cells (Figure 2C). We also determined if the level of other anti-apoptotic BCL-2 family members, BCL-2 and BCL-XL, thewere affected by flavopiridol treatment. No significant changes in BCL-XL were observed in any of the cells treated with flavopiridol (Figure 2C). However, we did detect a 2- and 2.5- fold decrease in BCL-2 protein levels in SaOS-2 and SJSA-1, respectively (Figure 2C). Altogether, flavopiridol decreased the protein levels of anti-apoptotic BCL-2 family members in all osteosarcoma cell lines, except U2OS. As mentioned before, previous reports have associated flavopiridol-induced apoptosis with an upregulation of E2F1, resulting in the transcriptional repression of MCL-1 [29, 30]. Consistent with these results, we observed a reduction in MCL-1 protein levels upon flavopiridol treatment in some of the osteosarcoma cell lines; however, E2F1 protein levels were unaffected in all the osteosarcoma cell lines analyzed (Figure 2D).

### Early effects of flavopiridol induce necrosis and G1 or G2/M arrest in osteosarcoma

**Figure 3:**
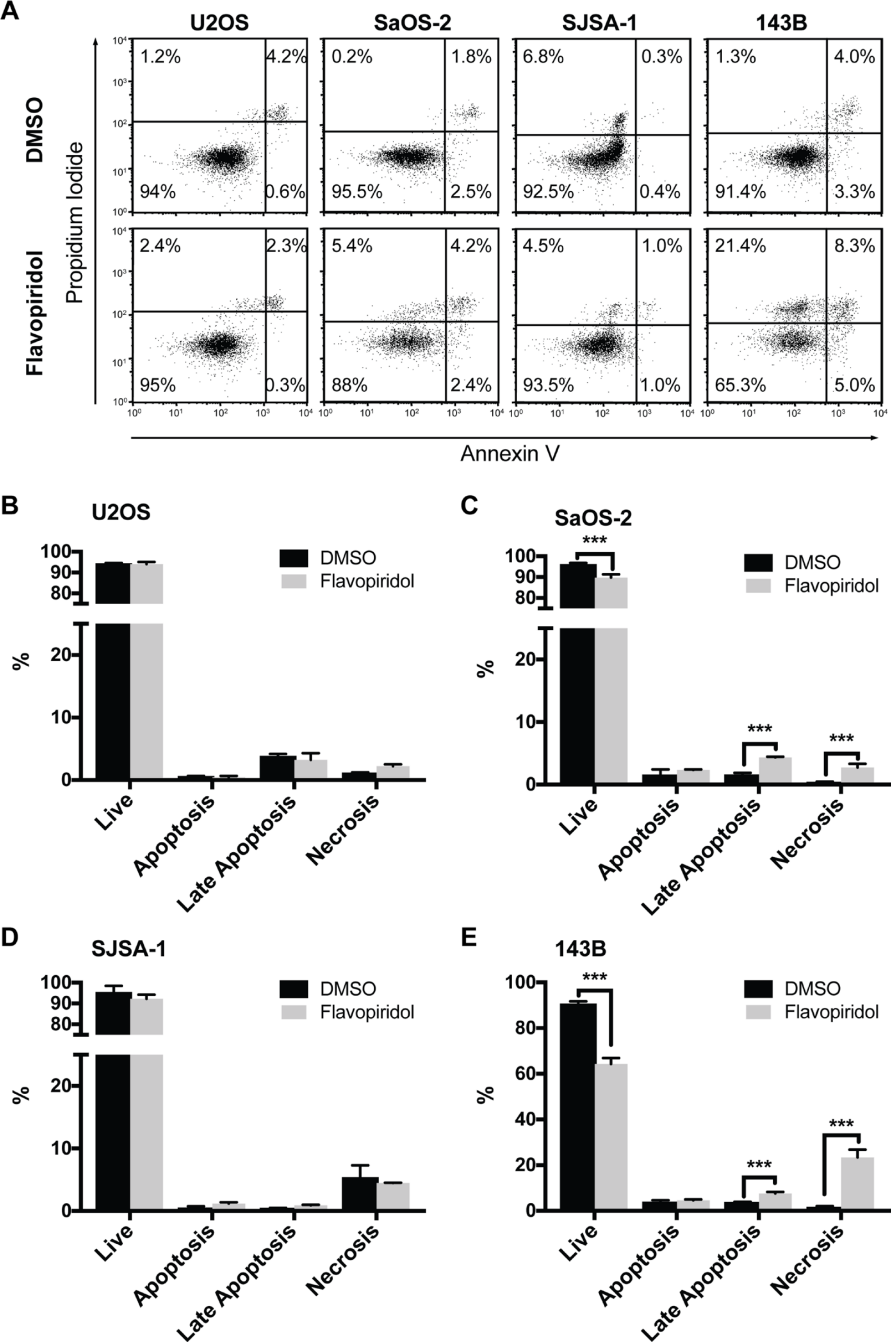
Increased necrosis after flavopiridol treatment in p53-null osteosarcomas Flow cytometry apoptosis analysis of osteosarcoma cell after treatment with 150 nM flavopiridol for 24 h. (**A**) Representative images of propidium iodide and Annexin V staining intensities observed in flavopiridol treated and DMSO control cells. (**B–E**) Quantification of the percentage of live, early apoptotic, late apoptotic, and necrotic cells for each osteosarcoma cell line. Each data point is mean ± s.d. of triplicate samples. ^***^p < 0.0002 by two-tailed *t* test.

We next examined if the decrease in protein expression of anti-apoptotic BCL-2 family members (MCL-1 and BCL-2) observed after treatment with flavopiridol (Figure 2) resulted in increased apoptosis. For this, we analyzed Annexin V levels and propidium iodide permeability by flow cytometry. Cells were treated with DMSO or 150 nM flavopiridol for 24 h and stained for Annexin V. In all the cell lines examined, we detected no changes in the number of apoptotic cells early after flavopiridol exposure (Figure 3A). Modest increase of 2.7 ± 0.3% and 3.8 ± 0.7% in late apoptosis and 2.4 ± 0.6% and 21.8 ± 3.4% increase in necrosis were observed in SaOS-2 and 143B, respectively (Figure 3C and 3E). No detectable changes in cellular viability were observed for U2OS and SJSA-1 within 24 h treatment with flavopiridol (Figure 3B and 3D).

**Figure 4:**
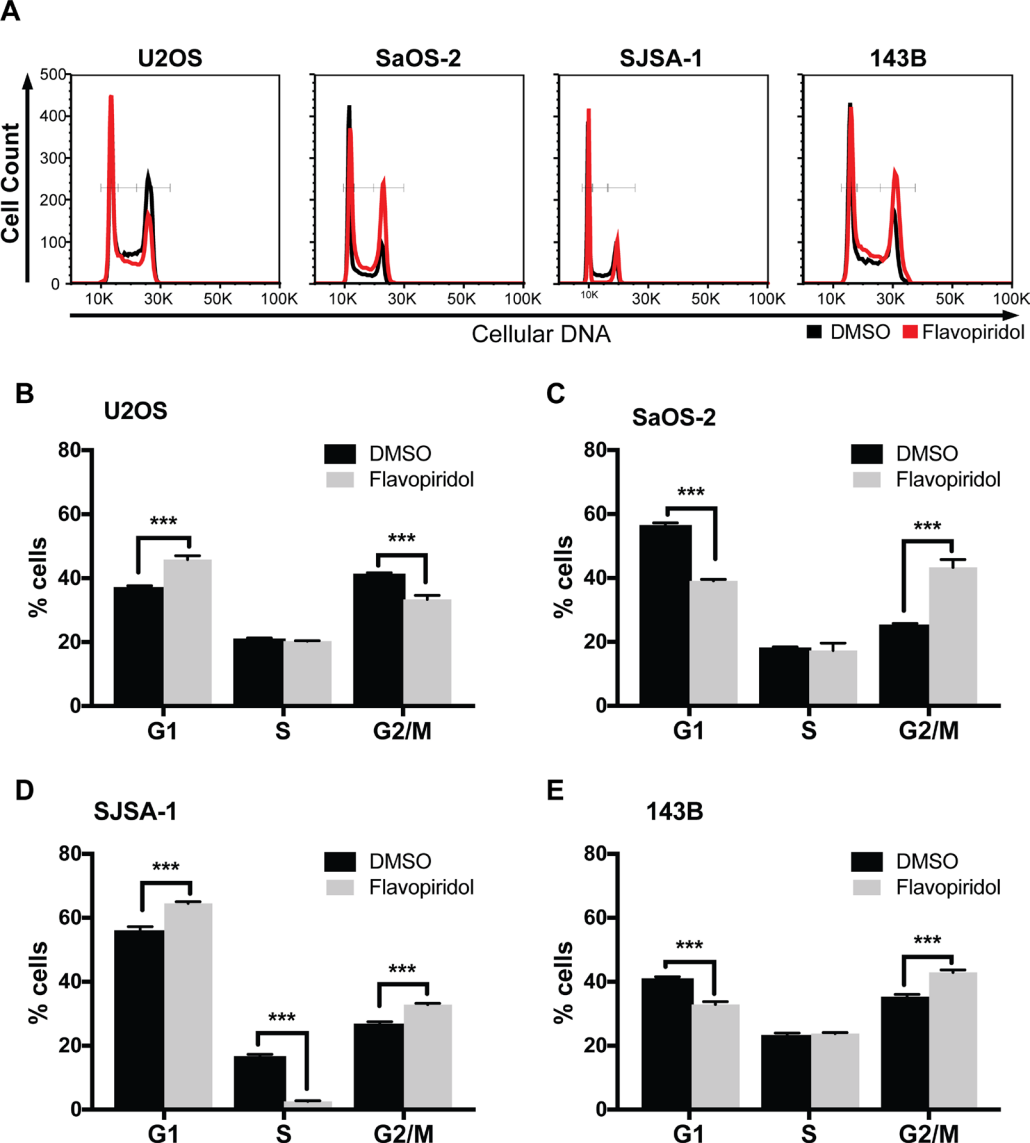
Cell cycle alterations after treatment of osteosarcomas with flavopiridol Flow cytometry cell cycle analysis of osteosarcoma cell after treatment with 150 nM flavopiridol for 24 h. (**A**) Representative images of propidium iodide staining intensity observed in flavopiridol treated and DMSO control cells. (**B–E**) Quantification of the percentage of cells in G1, S, and G2/M phase for each osteosarcoma cell line. Each data point is mean ± s.d. of triplicate samples. ^***^p < 0.0002 by two-tailed *t* test.

In parallel, we assessed cell cycle distributions and found that 150 nM flavopiridol resulted in a moderate proportional increase in G1 (median increase 8.5 ± 0.1%), with corresponding decreases in S phase in U2OS and SJSA-1 cells. In SaOS-2, SJSA-1, and 143B cells, flavopiridol treatment resulted in a moderate increase in G2/M (median increase 7.7 ± 6.5%), with a corresponding decrease in G1 and/or S phases (Figure 4).

### RNA-seq transcriptome analysis of flavopiridol treated osteosarcoma

**Figure 5:**
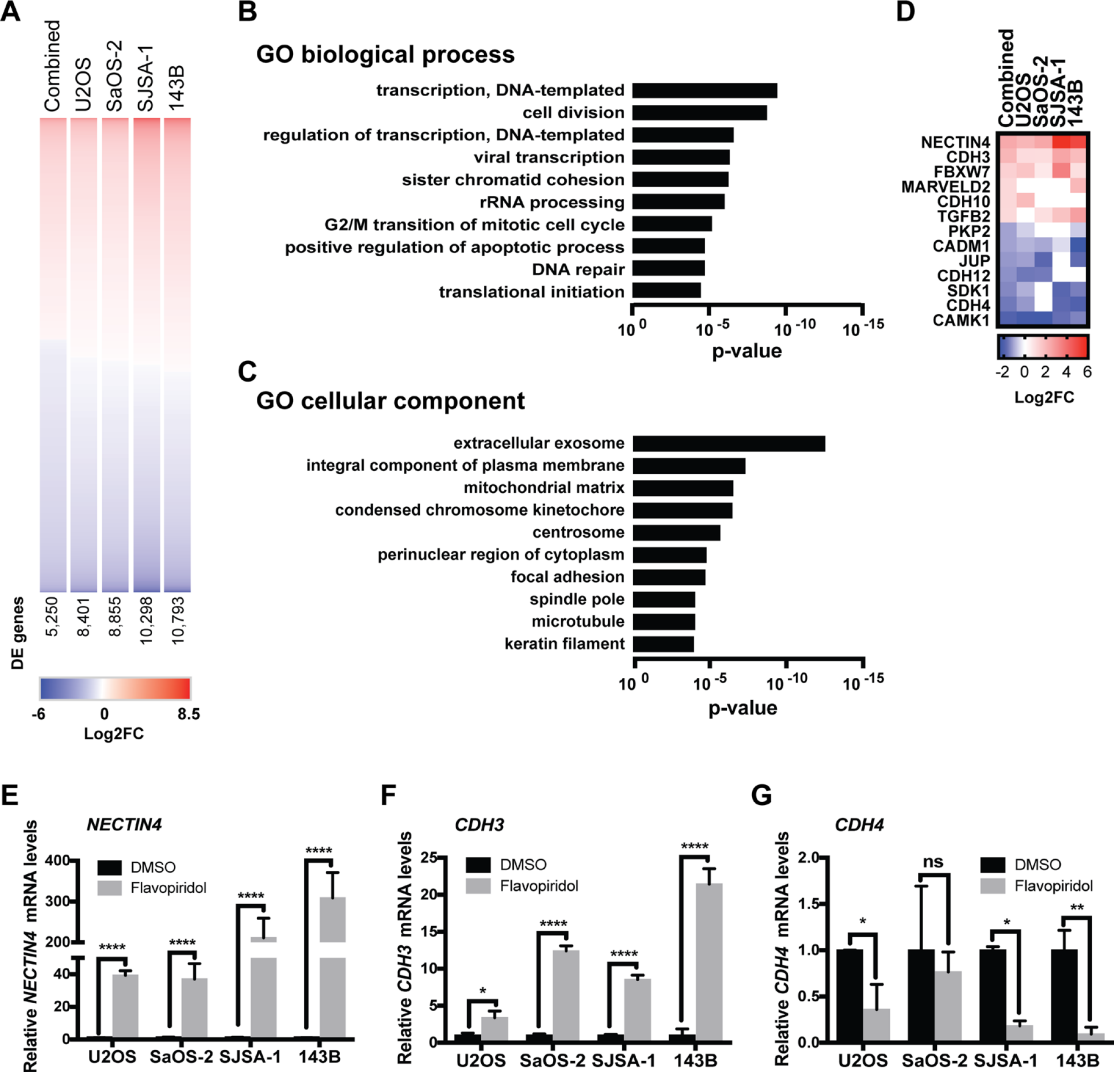
RNA-sequencing analysis of osteosarcoma cells treated with flavopiridol (**A**) Heatmap of differentially expressed (DE) genes in osteosarcoma cells treated with 150 nM flavopiridol for 24h. Number of DE genes shown. (**B–C**) Gene ontology (GO) enrichment analysis for biological processes (B) and cellular component (C) of the 1,820 genes with >2-linear-fold expression in the combined data set. (**D**) Heatmap of the DE genes involved in adherens junction assembly, maintenance and regulation. (**E–G**) RT-qPCR validation of some of the genes involved in cell adhesion: (E) *NECTIN4,* (F) *CDH3* and (G) CDH4. ^*^p < 0.0332, ^**^p < 0.0021, ^***^p < 0.0002, ^****^p < 0.0001, n.s. not significant by two-tailed *t* test.

To further investigate the effects of flavopiridol and its mechanism of action in osteosarcoma, we performed global gene expression profiling. Samples were treated in triplicate with DMSO or 150 nM flavopiridol for 24 h. Despite the known role of CDK7/9 in transcriptional elongation, flavopiridol treatment led to gene upregulation and downregulation in similar proportions (Figure 5A). We observed profound gene expression changes with a median 35.9 ± 9.0% of genes down- or up-regulated >2-linear-fold in osteosarcoma (2,282 out of 8,401 genes in U2OS, 2,631 out of 8,855 genes in SaOS-2, 4,688 out of 10,298 genes in SJSA-1, and 4,553 out of 10,793 genes in 143B). Combined analysis demonstrated a significant (5,250 genes) overlap of down- and upregulated genes across the four cell lines, indicating a common gene expression response to flavopiridol (Figure 5A).

To identify candidate mechanisms of action of flavopiridol in osteosarcoma, the 1,820 differentially-expressed genes with >2-linear-fold expression in the combined data set were subjected to gene ontology enrichment using PANTHER classification system for biological process and cellular component (Figure 5B and 5C and Supplementary Tables 1 and 2) [32]. As expected, gene sets related to regulation of transcription and cell division were enriched among flavopiridol-responsive genes. In addition, we observed enrichment of genes involved in focal adhesion. Further analysis of the differentially expressed genes data set pointed at an enrichment of genes involved in adherens junction assembly, maintenance and regulation (Figure 5D). We validated the top deregulated genes using real-time qPCR in independently generated samples and were able to confirm that genes potentially involved in cell migration – including *NECTIN4*, cadherin 3 (*CDH3*), cadherin 4 (*CDH4*), myocardin (*MYOCD*), mitogen-activated protein kinase 6 (*MAP2K6*), and FAT atypical cadherin 2, (*FAT2*) – are responsive to flavopiridol treatment (Figure 5E–5G and Supplementary Figure 1).

### Flavopiridol treatment reduces cell migration, invasion and metastasis in osteosarcoma

**Figure 6:**
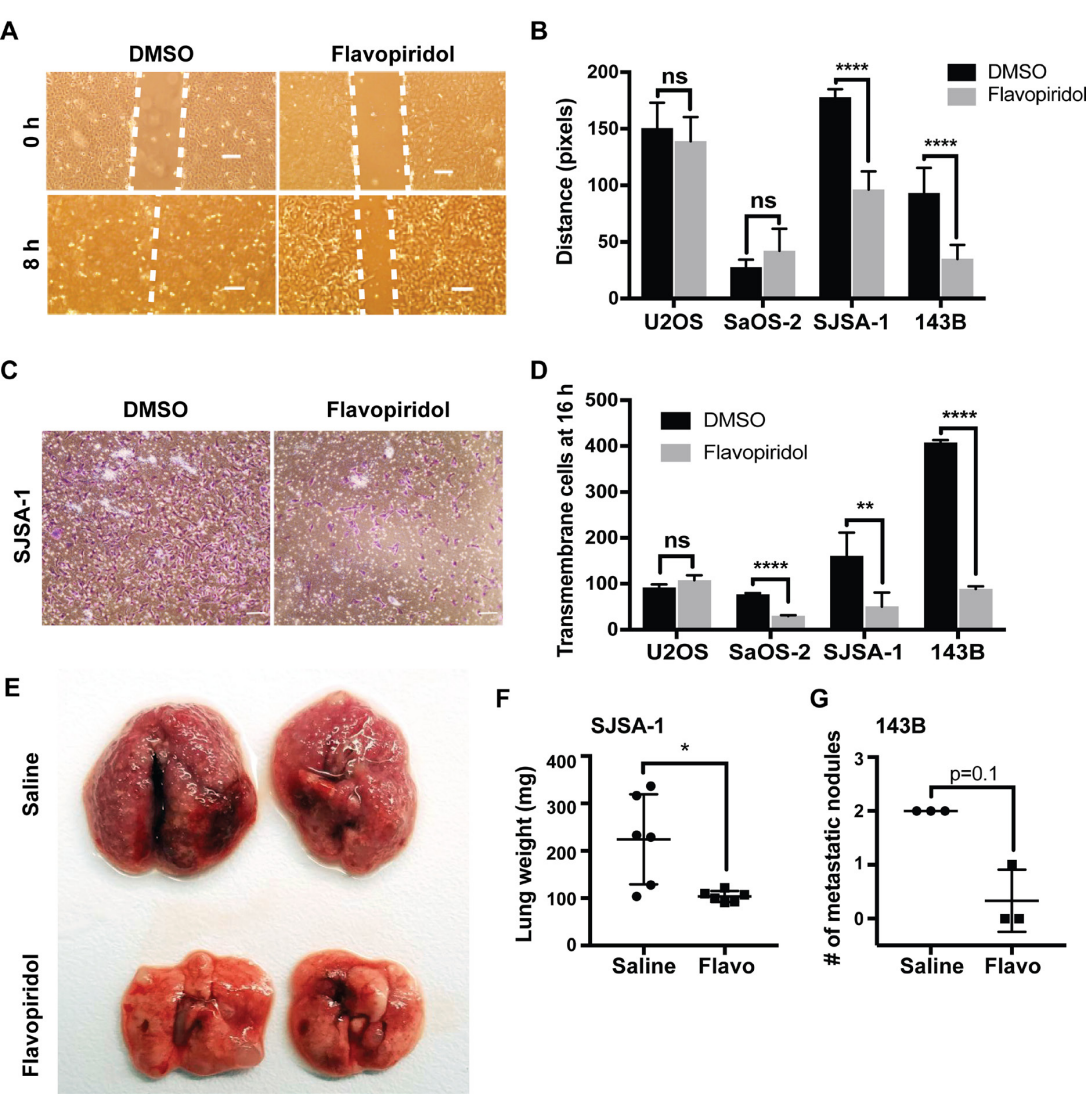
Flavopiridol reduces osteosarcoma cell migration, invasion and metastasis (**A–B**) Scratch-wound healing assay in osteosarcoma cells treated with 150 nM flavopiridol for a total of 24 h. Wound was allowed to heal for 8 h. (A) Representative image of control (DMSO) and treated SJSA-1 cells. Scale bar = 150 μm. (B) Quantification of the distance (pixels) migrated for each of the osteosarcoma cell lines. Each data point is mean ± s.d. of ten measurements in triplicate samples. (**C–D**) Chemotaxis cell invasion assay in osteosarcoma cells treated with 150 nM flavopiridol for 16 h. (C) Representative image for control (DMSO) and treated SJSA-1 cells. Scale bar = 150 μm. (D) Quantification of the number of cells that invaded the lower chamber after 16 h. (**E–G**) SJSA-1 and 143B lung colonization in athymic mice following tail vein injection and treated for 21 days with 2.5 mg/kg flavopiridol. (E) Representative images of lungs from SJSA-1 injected mice. (F) Quantification of the lung weight in mice injected with SJSA-1 cells. Each data point is mean ± s.d. of six lungs from three mice. (G) Quantification of the number of metastatic nodules in mice injected with 143B cells. Each data point is mean ± s.d. from three mice. ^*^p < 0.0332, ^**^p < 0.0021, ^****^p < 0.0001, n.s. not significant by two-tailed *t* test.

Since a significant number of genes involved in cellular adhesion were identified as flavopiridol-responsive genes, we evaluated if these changes translated into a functional effect of flavopiridol on cell migration, invasion, and metastasis. The low levels of cell death observed in response to flavopiridol within the first 24 h post-treatment presented an advantage for an advantage for analysis of migration data. First, we performed a scratch-wound healing assay in all four osteosarcoma cell lines treated with DMSO or 150 nM flavopiridol for a total of 24 h to assess changes in migration potential. We detected significant reduction in the ability of SJSA-1 and 143B cells to heal the wound within 8 h (Figure 6A–6B and Supplementary Figure 2). No significant changes were observed in cell migration in U2OS and SaOS-2 cells upon flavopiridol treatment. We then assessed chemotaxis cell invasion using a Transwell insert and bFGF as the chemo-attractant in the lower chamber. Cells in the upper chamber were treated with DMSO or 150 nM flavopiridol for 16 h. Consistent with our scratch-wound assay results, a significant lower number of SJSA-1 and 143B cells were able to invade to the bottom of the Transwell membrane when exposed to flavopiridol. Interestingly, we also observed significant fewer cells invading into the lower chamber in flavopiridol treated SaOS-2 (Figure 6C–6D and Supplementary Figure 3). No significant changes were observed in U2OS cells. As final validation, we examined whether flavopiridol could reduce the ability of osteosarcoma cells to metastasize into the lungs *in vivo*. For this, we tested the two most responsive cell lines, SJSA-1 and 143B. After injecting the cells into the tail vein of athymic mice, mice received daily intraperitoneal injections of saline or 2.5 mg/kg flavopiridol for 21 days. After 21 days, mice were euthanized and the burden of lung metastasis was evaluated. Consistent with the highly metastatic nature of the SJSA-1 cell line, saline treated mice had massive number of small metastatic nodules, which was significantly reduced by treatment with flavopiridol (Figure 6E–6F). The lungs of SJSA-1 saline-treated mice weight 2–3 times more than fflavopiridol-treated. A reduction in the total number metastatic nodules was also observed in flavopiridol-treated mice injected with 143B cells (Figure 6G). Significant weight loss was not observed in any of the six flavopiridol-treated mice.

## DISCUSSION

Poor response to chemotherapy is a major challenge in osteosarcoma treatment [3, 4]. Resistance to chemotherapy, likely a consequence of the overexpression of anti-apoptotic BCL-2 family members – including MCL-1, along with a high rate of metastasis are common problems encountered in osteosarcoma patients [7]. Previous studies trying to address some of these problems have shown promising results using CDK inhibitors to induce apoptosis in osteosarcoma cells *in vitro* [8]. Flavopiridol is a broad CDK inhibitor, known to effectively decrease MCL-1 and exhibiting promising activity in pre-clinical and clinical trials [14–16, 18]. In addition to its anti-proliferative activity, flavopiridol has pleiotropic biological effects including pro-apoptotic and anti-angiogenic activity that are likely due to its indirect action in reducing the levels of several cellular proteins [33]. These promising preclinical observations paved the way for phase I and phase II clinical trials as single agent in various malignant neoplasms (reviewed by Zhai *et al.* [34] and more recently as combination, consolidation, and maintenance therapy [15, 17]. These observations led us to evaluate the effect of flavopiridol in osteosarcoma.

In agreement with previous studies, our results show that flavopiridol exerts its anti-tumor activities independent of the type of driver mutations [35, 36]. We observed that flavopiridol was effective at decreasing the viability of U2OS, SaOS-2, SJSA-1, and 143B, four genetically different osteosarcoma cell lines of human origin, at nanomolar concentrations (Figure 1). The mechanisms underlying this phenomenon seem likely to be multifactorial, leading to the downregulation of MCL-1 and other BCL-2 family members (Figure 2C). While other studies have proposed that an increase in E2F1 expression could mediate the transcriptional repression of MCL-1 [29, 31], we were unable to detect changes in E2F1 protein levels upon flavopiridol treatment in osteosarcoma (Figure 2D). Our RNA-seq data identified F-box/WD repeat-containing protein 7 (*FBXW7*) as one of the genes differentially upregulated following treatment of flavopiridol. FBXW7 acts as a tumor suppressor involved in the ubiquitination and degradation by the proteasome of substrates with oncogenic activity, including MCL-1 [37]. Thus, it is possible that flavopiridol-mediated up-regulation of FBXW7 may be, at least in part, responsible for the decrease in MCL-1 protein levels observed in some osteosarcoma cell lines. Previous studies have also indicated that flavopiridol can mediate its cytotoxic effects through inhibition of CDK9 and CDK7, hence hampering global transcription [19, 20]. While we did not detect a predominance of downregulated genes upon flavopiridol treatment in our RNA-seq analysis, we did observe that the most significant changes across all four osteosarcoma cell lines studied were in genes associated with transcription regulation (Figure 5B).

We also examined the effect of flavopiridol treatment in osteosarcoma cell cycle progression. We observed that osteosarcoma cells treated with flavopiridol presented an increase in G0/G1, G2/M, or both. The S-phase was mostly unaffected, with a significant reduction observed only in SJSA-1 (Figure 4D). CDKs drive the cell cycle, so our results are in line with other studies showing similar effects with flavopiridol in other tumor cells and with the use of other CDK inhibitors [38]. These alterations in the cell cycle were also captured in our analysis of differentially expressed genes (Figure 5). Interestingly, our cell cycle results correlate with the genetic profile of the osteosarcoma cell lines. U2OS and SJSA-1, two cell lines with wild-type *TP53,* undergo G1-arrest following flavopiridol treatment. On the other hand, SaOS-2 and 143B, *TP53*-mutant cells, undergo G2/M arrest (Figure 4). Studies have shown that G2/M arrest can be associated with mitotic catastrophe, followed by cell death, depending on the p53 status of the cells [39, 40]. SaOS-2 and 143B not only presented G2/M arrest but also displayed increased necrosis, suggesting that flavopiridol induces mitotic catastrophe in these *TP53*-null cells.

The results from our RNA-seq analyses revealed that a significant number of flavopiridol-sensitive transcriptional alterations occur in genes coding for proteins involved in cell-cell and cell-matrix interactions. Some of these changes that best correlated with the functional changes in cell migration, were upregulation of *CDH3*, the gene coding for P-cadherin and downregulation of *CDH4,* the gene coding for R-cadherin. In the absence of E-cadherin (*CDH1*) expression, P-cadherin is able to suppress invasion by its strong interaction with catenins, similar to E-cadherin in cell-cell adhesion [41]. In epithelial breast cells (MCF10A), it has been suggested that P-cadherin is involved in increasing the levels of intercellular tension within cells, whereas E-cadherin dictates the rate at which the intercellular tension builds over time. In the absence of E-cadherin, P-cadherin takes over the role as tension regulator, triggering mechanotransduction and preventing a decrease in intercellular tension, thus acting as suppressor of cancer invasion [42]. Consistent with this model, and in line with previous reports describing a lack of E-cadherin and P-cadherin expression in human osteosarcoma, our transcriptome data shows no baseline *CDH1* or *CDH3* expression in the osteosarcoma cell lines analyzed in this study. Thus, the increase in *CDH3* expression following flavopiridol treatment may potentially suppress invasion in osteosarcoma cells (Figure 5E). Further, we also observed a downregulation of the R-cadherin (*CDH4*) transcript in all flavopiridol-treated cell lines (Figure 5G). A recent study showed that R-cadherin is overexpressed in SaOS-2, SJSA-1, and U2OS, and that the knock-down of *CDH4* by shRNA in U2OS reduces migration and invasion of these osteosarcoma cell lines [43]. Thus, flavopiridol indirect induction of P-cadherin and concomitant inhibition of R-cadherin expression is likely to contribute to its anti-metastatic activity in osteosarcoma.

Functional analyses of the transcriptional changes observed in flavopiridol-treated cells, clearly indicate striking effects of this drug in reducing cell migration, invasion and metastasis in osteosarcoma (Figure 6). Overall, we identified that SJSA-1 and 143B were particularly sensitive to the therapeutic effects of flavopiridol. Interestingly, while we observed a significant reduction in the number of metastases in flavopiridol treated mice; the few metastases that were able to home and grow in the lung were not smaller in size. This might indicate that flavopiridol can decrease the cells ability to metastasize, but has no effect on the cells’ growth once they have homed into the lung. Similar to what has been observed in other cancers, the use of flavopiridol for osteosarcoma therapeutics might work better in combination with other cytotoxic agents. Further experiments are needed to determine which gene or gene combination changes are functionally relevant for the decreased migration, invasion, and metastasis observed upon flavopiridol treatment.

CDK inhibitors, including flavopiridol, are known to induce apoptosis in many tumor cell types; however, this study shows for the first time that flavopiridol can effectively inhibit tumor migration, invasion, and metastasis in osteosarcoma. We suggest that flavopiridol, likely in combination with other cytotoxic chemotherapeutic agents, may be a promising strategy for treatment of bone tumors. Beyond its ability to kill osteosarcoma cells *in vitro*, it decreases cell migration and invasion both *in vitro* and *in vivo*. It is effective at low concentrations and active within concentrations that were found to be tolerable in phase I clinical trials [15, 17].

## MATERIALS AND METHODS

### Cell culture

Osteosarcoma cell lines U2OS, SaOS-2, SJSA-1, and 143B were purchased from the American Type Culture Collection (ATCC). Cells were cultured in a humidified atmosphere at 37° C and 5% CO_2_. U2OS were cultured in McCoy's 5A Modified Medium (Gibco) supplemented with 10% BCS (Sigma) and 1% streptomycin/penicillin (Gibco). SaOS-2 were cultured in McCoy's 5A Modified Medium (Gibco), supplemented with 15% BCS (Sigma) and 1% streptomycin/penicillin (Gibco). SJSA-1 were cultured in RPMI 1640 (Gibco), supplemented with 10% BCS, 1% streptomycin/penicillin (Gibco) and 1% GlutaMAX (Gibco). 143B were cultured in MEM (Gibco), supplemented with 10% BCS, 1% streptomycin/penicillin (Gibco), 0.015 mg/ml BrdU (Sigma), and 1% GlutaMAX (Gibco). Flavopiridol (Alvocidib) was purchased from Selleck Chemicals (Houston, TX, USA).

### Cell viability assay

All cells were seeded into 96-well black assay plates (Costar) at a density of 1,000 cells per well. Flavopiridol was added 24 h after plating at concentrations ranging from 0 to 1 μM. Following treatment, cell viability was determined at every 24 h between 24–96 h post-treatment using CellTiter-Glo (Promega) according to manufacturer's instructions. Dose response curves were generated using GraphPad Prism.

### Real time RT-PCR

Real-time RT-PCR experiments were performed using the 7500 real-time PCR System (Applied Biosystems). Primers were designed using Integrated DNA Technologies software. RNA was prepared using Trizol, and cDNA was synthesized using the Superscript system (Invitrogen). Samples were analyzed in triplicate and normalized to GAPDH expression levels. 500 ng of sample RNA was used for cDNA synthesis (Superscript III First-Strand Synthesis System) and SYBR-Green Master Mix (Applied Biosystems). Primer sequences: *CDH3*: hCDH3-F 5′-ATGA CGTGGCACCAACCAT-3′ and hCDH3-R 5′-GTTAGCCG CCTTCAGGTTCTC-3′; *Nectin-4*: hNectinExon3-F 5′- ACTGACTTGTGTGGTGTCC-3′ and hNectinExon4-R 5′-ATAGCTCCTTCTCTGCCAATG-3′; *CDH4*: hCDH4-F 5′-GCTGTGTCCTTAGTGCTGTTAG-3′ and hCDH4-R 5′-GTGAAGACAGAGTGCCTCTTG-3′; *MAP2K6*: hMAP2K6-F 5′- CACCTTTTATGGCGCACTGTT-3′ and hMAP2K6-R 5′-TCCATGAGCTCCATGCAGATC-3′; *MYOCD*: hMYOCD-F 5′-CCAAAGTTTTCAATTCCATCC CC-3′ and hMYOCD-R 5′-CTTTCAATAAGCACGTCC AGG-3′; *FAT2*: hFAT2-F 5′- GTTGTCCCTTGAAATGTG CTC-3′ and hFAT2-R 5′-GACCCTAGTGCTGTTTCTGG -3′; *MCL-1*: hMCL-1F 5′-TCAATTCCTACAGCTTTCCC C-3′ and hMCL-1R 5′-GGGTTTCACAGTGCCAAAA TC-3′; *18S*: h18SF 5′-GTAACCCGTTGAACCCCATT-3′ and h18SR 5′-CCATCCAATCGGTAGTAGCG-3.

### Western blotting

Western blotting was performed as previously described [44]. Cells were seeded into 6 well plates, cultured for 24 h and treated as indicated. Briefly, cells were lysed for 30 min on ice in RIPA buffer (65 mM Tris pH 7.4, 150 mM NaCl, 1% NP-40, 0.25% sodium deoxycholate, 1 mM EDTA) containing protease inhibitor cocktail (Roche Diagnostics, IN, USA). Lysates were cleared by centrifugation at 14000 RPM at 4° for 20 min. Protein concentration from cell lysate was measured by using a BCA protein assay kit (Pierce). 20 μg of total protein were resolved in 4–15% SDS-PAGE gel (Bio-Rad) and transferred to PDVF membrane (Millipore). Non-specific binding was prevented blocking the membrane with 3% non-fat dry milk in TBS-0.1% Tween (TBS-T) for 1 h at RT. Membranes were incubated at 4° C overnight in primary antibody: 1:1000 anti-human MCL-1 (4572S, Cell Signaling), 1:2000 anti-human tubulin (2144S, Cell Signaling), 1:1000 anti-mouse E2F1 (3742S, Cell Signaling), 1:5000 anti-actin (A1978, Sigma), 1:1000 anti-mouse Bcl-2 (15071, Cell Signaling), and 1:1000 anti-rabbit Bcl-XL (2764, Cell Signaling). Membranes were rinsed 3 times for 5 min with TBS-T on shaker and incubated with anti-mouse or anti-rabbit IgG conjugated to horseradish peroxidase (Vector Laboratories) 1:1000 for 1 h at room temperature. Following 3 washes with TBS-T for 5 min, protein signals were detected using the Super Signal West Dura Extended Duration Substrate (Pierce).

### Apoptosis analysis

1 × 10^6^ cells were seeded in 100 mm plates. 24 h after seeding, cells were treated with 150 nM flavopiridol or DMSO as control. After 24 h treatment, all cells were collected, counted and resuspended in Annexin V binding buffer (BioLegend) at a concentration of 4 × 10^6^ cells/ml. We mixed 50 μl of cell suspension with 2.5 μl of APC Annexin V (BioLegend) along with 50 μg/ml of propidium iodide and incubated 15 min at room temperature in the dark. Subsequently, 200 μl of Annexin V binding buffer was added into each sample and immediately analyzed by flow cytometry. Flow cytometry analyses of the cells were performed with the BD FACS Calibur, LSRII or Attune Acoustic Focusing Cytometer.

### Cell cycle analysis

1 × 10^6^ cells were seeded in a 100 mm plate. 24 h after seeding, cells were treated with 150 nM flavopiridol or DMSO as control. After 24 h treatment, cells were trypsinized, counted and fixed with 70% ethanol overnight. Subsequently, 50 μg of RNase was added to each sample for 30 minutes at 37° C before addition of 5 μg/sample of propidium iodide (Sigma). Flow cytometry analyses of the cells were performed with the Attune Acoustic Focusing Cytometer and data analysis was performed using FlowJo software.

### Molecular index library preparation and RNA sequencing

Total RNA was isolated using the RNA Micro Kit (Qiagen). Subsequently, 500 ng of total RNA was used to create the RNA-seq library following the manufacturer's protocol from purification, mRNA fragmentation through the adenylation of end-repaired cDNA fragments and cleanup (TruSeq Stranded mRNA, Illumina). The collected sample was cleaned with AMPure XP beads (Beckman Coulter) and eluted in 20 μl of 10 mM Tris buffer, pH 8, 0.1% Tween 20. A paired-end 100-bp sequencing run was performed on HiSeq 4000 yielding 348M PE reads with a final library concentration of 2 nM as determined by qPCR (KAPA Biosystem). Statistical analysis for differential gene expression was performed using DESeq2.

### Scratch-wound healing assay

Cells (1 × 10^6^/well) were plated on 6-well plates. The day after, once cells were grown to a confluence of about 80%, 150 nM flavopiridol or DMSO (vehicle control) was added to the monolayer. 16 h after treatment, the monolayer was scratched using a 10 μl sterile pipette tip. Images were captured immediately and 8 h after the wound (24 h post-flavopiridol treatment). The exact location of the image was marked to identify the same gap. The distances between the boundaries of the wound at 0 and 8 h at 10 different locations were measured in pixels using Zen software (Zeiss).

### Transwell migration assay

PET membrane permeable support inserts for 24 well plates (Falcon) were pretreated with Matrigel (25 μg/insert, Corning), resuspended in DMEM media with no supplements. 2 × 10^4^ cells were seeded onto the Matrigel-coated membrane. FGF-2 (EMD Millipore) was added as chemo-attractant in the bottom chamber (100 ng/ml). Flavopiridol (150 nM) or DMSO was added to the upper chamber. Cells were incubated in a humidified atmosphere at 37° C and 5% CO_2_ for 16 h. After 16 h, cells that passed through the Matrigel-coated membrane were fixed and stained in a solution containing 0.5% crystal violet (Sigma) and 6% glutaraldehyde (Sigma). Images were captured. Crystal violet was dissolved in 300 μl of 33% acetic acid and the absorbance at 573 nm was measured.

### Mouse models

Athymic nude (NU/J) mice were obtained from The Jackson Laboratories. 2 × 10^6^ cells (SJSA-1 or 143B) were injected into the tail vein of the mice. For the following 21 days, mice received daily intra-peritoneal injections of saline or 2.5 mg/kg flavopiridol. After 21 days, mice were euthanized and lungs were collected for analysis. Metastatic burden was measured as number of metastatic nodules for the 143B cell line and individual lung weight was used for SJSA-1 due to high number of the metastatic nodules in the control group. The University of California Irvine Institutional Animal Care and Use Committee approved all animal procedures. Three animals were used for each group.

## SUPPLEMENTARY MATERIALS FIGURES AND TABLES







## References

[1] Ottaviani G, Jaffe N (2009). The epidemiology of osteosarcoma. Cancer Treat Res.

[2] Mirabello L, Troisi RJ, Savage SA (2009). Osteosarcoma incidence and survival rates from 1973 to 2004: data from the Surveillance, Epidemiology, and End Results Program. Cancer.

[3] He H, Ni J, Huang J (2014). Molecular mechanisms of chemoresistance in osteosarcoma (Review). Oncol Lett.

[4] Chou AJ, Gorlick R (2006). Chemotherapy resistance in osteosarcoma: current challenges and future directions. Expert Rev Anticancer Ther.

[5] Luetke A, Meyers PA, Lewis I, Juergens H (2014). Osteosarcoma treatment - where do we stand? A state of the art review. Cancer Treat Rev.

[6] Raymond AK, Chawla SP, Carrasco CH, Ayala AG, Fanning CV, Grice B, Armen T, Plager C, Papadopoulos NE, Edeiken J (1987). Osteosarcoma chemotherapy effect: a prognostic factor. Semin Diagn Pathol.

[7] Thallinger C, Wolschek MF, Maierhofer H, Skvara H, Pehamberger H, Monia BP, Jansen B, Wacheck V, Selzer E (2004). Mcl-1 is a novel therapeutic target for human sarcoma: synergistic inhibition of human sarcoma xenotransplants by a combination of mcl-1 antisense oligonucleotides with low-dose cyclophosphamide. Clin Cancer Res.

[8] Fu W, Ma L, Chu B, Wang X, Bui MM, Gemmer J, Altiok S, Pledger WJ (2011). The cyclin-dependent kinase inhibitor SCH 727965 (dinacliclib) induces the apoptosis of osteosarcoma cells. Mol Cancer Ther.

[9] Losiewicz MD, Carlson BA, Kaur G, Sausville EA, Worland PJ (1994). Potent inhibition of CDC2 kinase activity by the flavonoid L86-8275. Biochem Biophys Res Commun.

[10] Byrd JC, Shinn C, Waselenko JK, Fuchs EJ, Lehman TA, Nguyen PL, Flinn IW, Diehl LF, Sausville E, Grever MR (1998). Flavopiridol induces apoptosis in chronic lymphocytic leukemia cells via activation of caspase-3 without evidence of bcl-2 modulation or dependence on functional p53. Blood.

[11] Konig A, Schwartz GK, Mohammad RM, Al-Katib A, Gabrilove JL (1997). The novel cyclin-dependent kinase inhibitor flavopiridol downregulates Bcl-2 and induces growth arrest and apoptosis in chronic B-cell leukemia lines. Blood.

[12] Kansara M, Thomas DM (2007). Molecular pathogenesis of osteosarcoma. DNA Cell Biol.

[13] Chen X, Bahrami A, Pappo A, Easton J, Dalton J, Hedlund E, Ellison D, Shurtleff S, Wu G, Wei L, Parker M, Rusch M, Nagahawatte P (2014). Recurrent somatic structural variations contribute to tumorigenesis in pediatric osteosarcoma. Cell Rep.

[14] Bogenberger J, Whatcott C, Hansen N, Delman D, Shi CX, Kim W, Haws H, Soh K, Lee YS, Peterson P, Siddiqui-Jain A, Weitman S, Stewart K (2017). Combined venetoclax and alvocidib in acute myeloid leukemia. Oncotarget.

[15] Awan FT, Jones JA, Maddocks K, Poi M, Grever MR, Johnson A, Byrd JC, Andritsos LA (2016). A phase 1 clinical trial of flavopiridol consolidation in chronic lymphocytic leukemia patients following chemoimmunotherapy. Ann Hematol.

[16] Lanasa MC, Andritsos L, Brown JR, Gabrilove J, Caligaris-Cappio F, Ghia P, Larson RA, Kipps TJ, Leblond V, Milligan DW, Janssens A, Johnson AJ, Heerema NA (2015). Final results of EFC6663: a multicenter, international, phase 2 study of alvocidib for patients with fludarabine-refractory chronic lymphocytic leukemia. Leuk Res.

[17] Hofmeister CC, Poi M, Bowers MA, Zhao W, Phelps MA, Benson DM, Kraut EH, Farag S, Efebera YA, Sexton J, Lin TS, Grever M, Byrd JC (2014). A phase I trial of flavopiridol in relapsed multiple myeloma. Cancer Chemother Pharmacol.

[18] Chen R, Keating MJ, Gandhi V, Plunkett W (2005). Transcription inhibition by flavopiridol: mechanism of chronic lymphocytic leukemia cell death. Blood.

[19] Morales F, Giordano A (2016). Overview of CDK9 as a target in cancer research. Cell Cycle.

[20] Yeh YY, Chen R, Hessler J, Mahoney E, Lehman AM, Heerema NA, Grever MR, Plunkett W, Byrd JC, Johnson AJ (2015). Up-regulation of CDK9 kinase activity and Mcl-1 stability contributes to the acquired resistance to cyclin-dependent kinase inhibitors in leukemia. Oncotarget.

[21] Hsin JP, Manley JL (2012). The RNA polymerase II CTD coordinates transcription and RNA processing. Genes Dev.

[22] Soner BC, Aktug H, Acikgoz E, Duzagac F, Guven U, Ayla S, Cal C, Oktem G (2014). Induced growth inhibition, cell cycle arrest and apoptosis in CD133+/CD44+ prostate cancer stem cells by flavopiridol. Int J Mol Med.

[23] Schrump DS, Matthews W, Chen GA, Mixon A, Altorki NK (1998). Flavopiridol mediates cell cycle arrest and apoptosis in esophageal cancer cells. Clin Cancer Res.

[24] Ponten J, Saksela E (1967). Two established *in vitro* cell lines from human mesenchymal tumours. Int J Cancer.

[25] Fogh J, Fogh JM, Orfeo T (1977). One hundred and twenty-seven cultured human tumor cell lines producing tumors in nude mice. J Natl Cancer Inst.

[26] Mahoney E, Lucas DM, Gupta SV, Wagner AJ, Herman SE, Smith LL, Yeh YY, Andritsos L, Jones JA, Flynn JM, Blum KA, Zhang X, Lehman A (2012). ER stress and autophagy: new discoveries in the mechanism of action and drug resistance of the cyclin-dependent kinase inhibitor flavopiridol. Blood.

[27] Wall NR, O'Connor DS, Plescia J, Pommier Y, Altieri DC (2003). Suppression of survivin phosphorylation on Thr34 by flavopiridol enhances tumor cell apoptosis. Cancer Res.

[28] Gojo I, Zhang B, Fenton RG (2002). The cyclin-dependent kinase inhibitor flavopiridol induces apoptosis in multiple myeloma cells through transcriptional repression and down-regulation of Mcl-1. Clin Cancer Res.

[29] Croxton R, Ma Y, Song L, Haura EB, Cress WD (2002). Direct repression of the Mcl-1 promoter by E2F1. Oncogene.

[30] Ma Y, Cress WD, Haura EB (2003). Flavopiridol-induced apoptosis is mediated through up-regulation of E2F1 and repression of Mcl-1. Mol Cancer Ther.

[31] Jiang J, Matranga CB, Cai D, Latham VM, Zhang X, Lowell AM, Martelli F, Shapiro GI (2003). Flavopiridol-induced apoptosis during S phase requires E2F-1 and inhibition of cyclin A-dependent kinase activity. Cancer Res.

[32] Mi H, Muruganujan A, Casagrande JT, Thomas PD (2013). Large-scale gene function analysis with the PANTHER classification system. Nat Protoc.

[33] Newcomb EW (2004). Flavopiridol: pleiotropic biological effects enhance its anti-cancer activity. Anticancer Drugs.

[34] Zhai S, Senderowicz AM, Sausville EA, Figg WD (2002). Flavopiridol, a novel cyclin-dependent kinase inhibitor, in clinical development. Ann Pharmacother.

[35] Heijkants R, Willekens K, Schoonderwoerd M, Teunisse A, Nieveen M, Radaelli E, Hawinkels L, Marine JC, Jochemsen A (2018). Combined inhibition of CDK and HDAC as a promising therapeutic strategy for both cutaneous and uveal metastatic melanoma. Oncotarget.

[36] Carlson BA, Dubay MM, Sausville EA, Brizuela L, Worland PJ (1996). Flavopiridol induces G1 arrest with inhibition of cyclin-dependent kinase (CDK) 2 and CDK4 in human breast carcinoma cells. Cancer Res.

[37] Inuzuka H, Fukushima H, Shaik S, Liu P, Lau AW, Wei W (2011). Mcl-1 ubiquitination and destruction. Oncotarget.

[38] Fu W, Sharma SS, Ma L, Chu B, Bui MM, Reed D, Pledger WJ (2013). Apoptosis of osteosarcoma cultures by the combination of the cyclin-dependent kinase inhibitor SCH727965 and a heat shock protein 90 inhibitor. Cell Death Dis.

[39] Mc Gee MM (2015). Targeting the Mitotic Catastrophe Signaling Pathway in Cancer. Mediators Inflamm.

[40] Vakifahmetoglu H, Olsson M, Zhivotovsky B (2008). Death through a tragedy: mitotic catastrophe. Cell Death Differ.

[41] Ribeiro AS, Sousa B, Carreto L, Mendes N, Nobre AR, Ricardo S, Albergaria A, Cameselle-Teijeiro JF, Gerhard R, Soderberg O, Seruca R, Santos MA, Schmitt F (2013). P-cadherin functional role is dependent on E-cadherin cellular context: a proof of concept using the breast cancer model. J Pathol.

[42] Bazellieres E, Conte V, Elosegui-Artola A, Serra-Picamal X, Bintanel-Morcillo M, Roca-Cusachs P, Munoz JJ, Sales-Pardo M, Guimera R, Trepat X (2015). Control of cell-cell forces and collective cell dynamics by the intercellular adhesome. Nat Cell Biol.

[43] Tang Q, Lu J, Zou C, Shao Y, Chen Y, Narala S, Fang H, Xu H, Wang J, Shen J, Khokha R (2018). CDH4 is a novel determinant of osteosarcoma tumorigenesis and metastasis. Oncogene.

[44] Benavente CA, Finkelstein D, Johnson DA, Marine JC, Ashery-Padan R, Dyer MA (2014). Chromatin remodelers HELLS and UHRF1 mediate the epigenetic deregulation of genes that drive retinoblastoma tumor progression. Oncotarget.

