# Supplementary material for: The cyclin-dependent kinase inhibitor flavopiridol (alvocidib) inhibits metastasis of human osteosarcoma cells
Source: Oncotarget. 2018 May 4;9(34):23505–18. doi: 10.18632/oncotarget.25239 (PMC5955096; doi:10.18632/oncotarget.25239)
Supplement: Supplementary file 1 [file oncotarget-09-23505-s001.pdf]

The cyclin-dependent kinase inhibitor flavopiridol (alvocidib) inhibits metastasis of human osteosarcoma cells

SUPPLEMENTARY MATERIALS

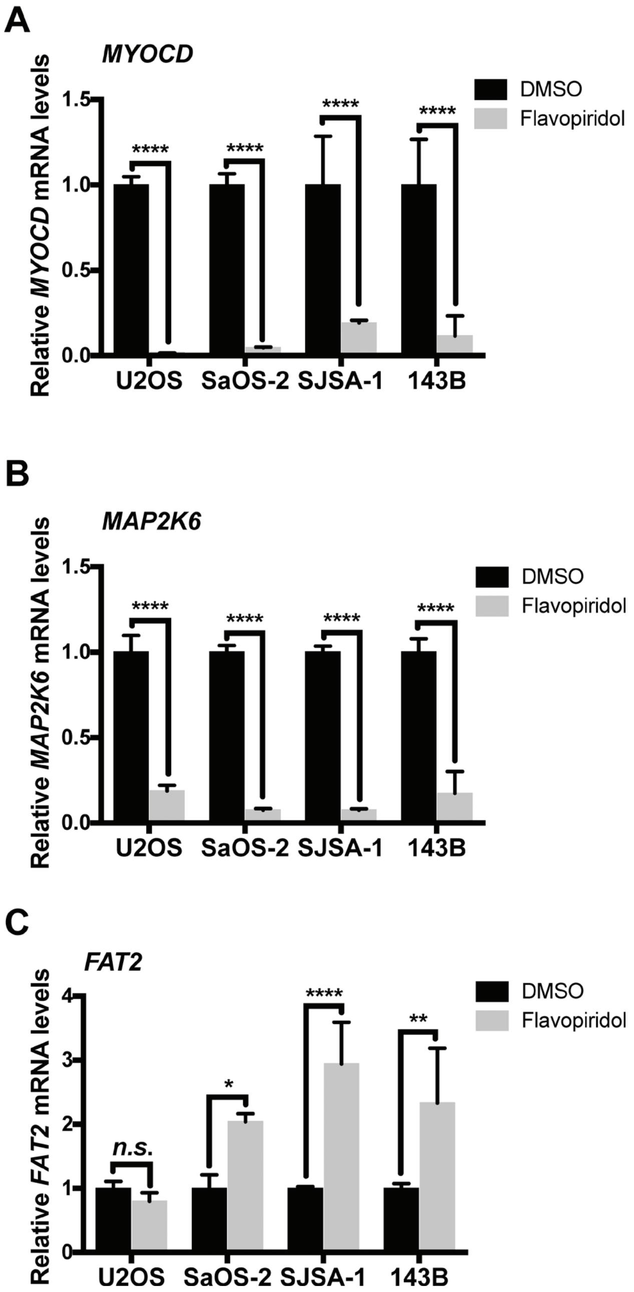

Supplementary Figure 1: RT-qPCR validation of genes involved in cell migration and tumor invasion. RT-qPCR validation of some of the genes involved in cell adhesion deregulated in osteosarcoma cells treated with 150 nM flavopiridol for 24 h. (**A**) *MYOCD*, (**B**) *MAP2K6*, and (**C**) *FAT2*.

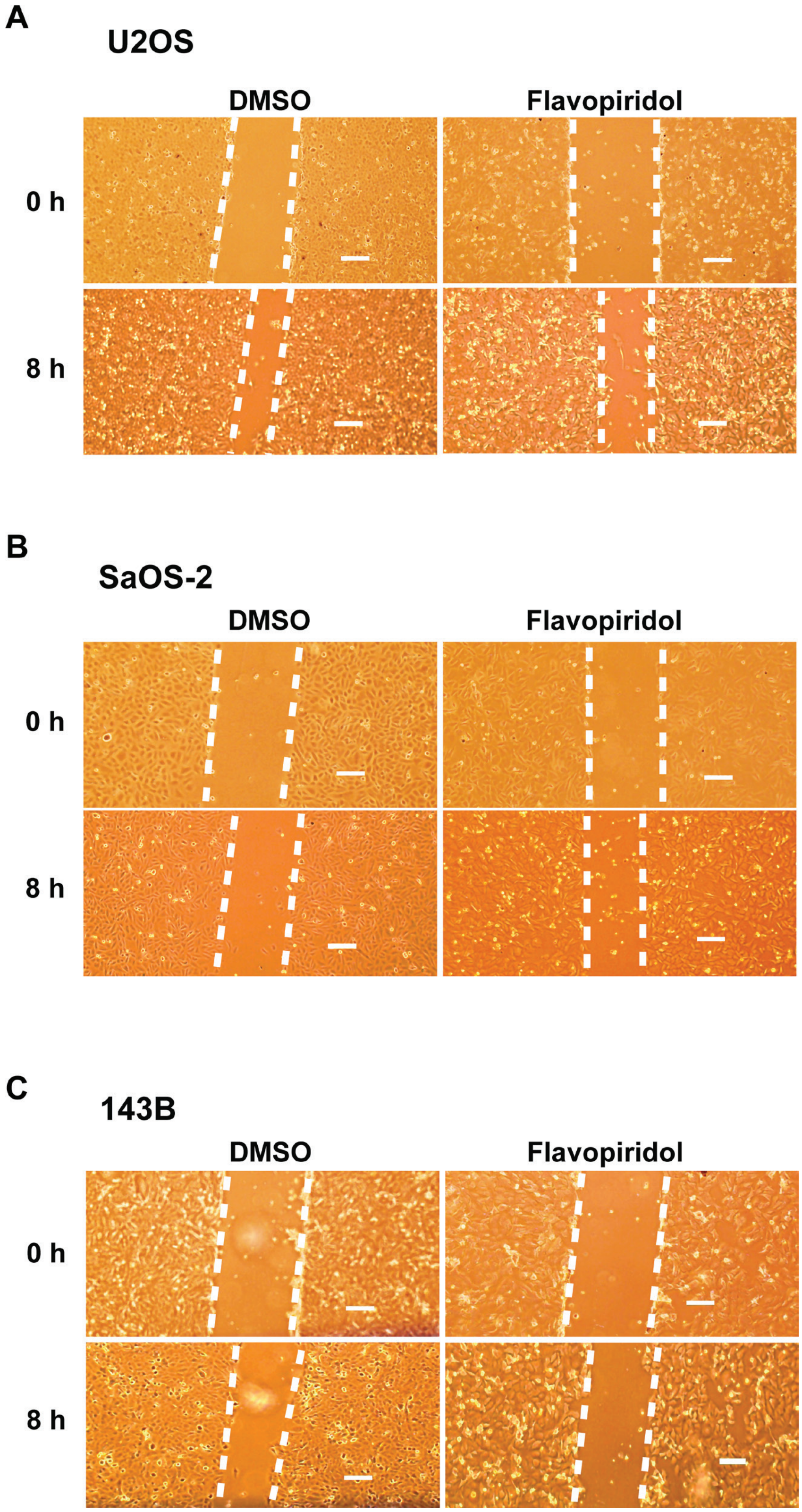

Supplementary Figure 2: Scratch-wound healing assay. Scratch-wound healing assay in osteosarcoma cells treated with 150 nM flavopiridol for a total of 24 h. Wound was allowed to heal for 8 h. (A–C) Representative images for control (DMSO) and treated (A) U2OS, (B) SaOS-2, and (C) 143B cells.

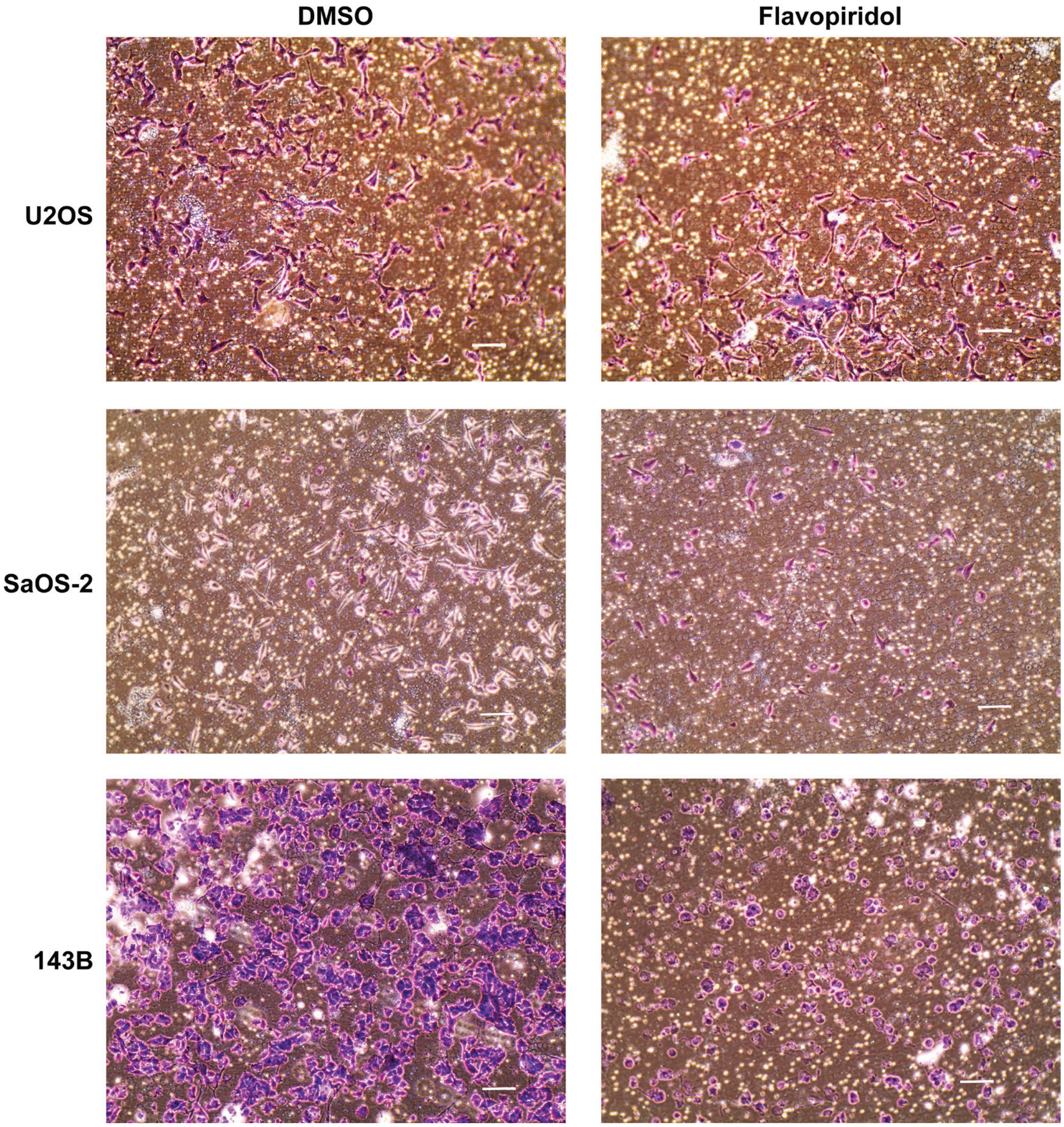

Supplementary Figure 3: Chemotaxis invasion assay. Chemotaxis cell invasion assay in osteosarcoma cells treated with 150 nM flavopiridol for 16 h. Representative image for control (DMSO) and treated U2OS, SaOS-2, and 143B cells.

Supplementary Table 1: RNA-seq gene ontology analysis for biological process. See Supplementary_Table_1

Supplementary Table 2: RNA-seq gene ontology analysis for cellular component. See Stupplementary_Table_2
